# Transcriptomic Analysis of the Kuruma Prawn *Marsupenaeus japonicus* Reveals Possible Peripheral Regulation of the Ovary

**DOI:** 10.3389/fendo.2020.00541

**Published:** 2020-08-19

**Authors:** Naoaki Tsutsui, Yasuhisa Kobayashi, Kouichi Izumikawa, Tatsuya Sakamoto

**Affiliations:** ^1^Department of Marine Bioresources, Faculty of Bioresources, Mie University, Tsu, Japan; ^2^Faculty of Science, Ushimado Marine Institute, Okayama University, Setouchi, Japan; ^3^Department of Fisheries, Faculty of Agriculture, Kindai University, Nara, Japan; ^4^Research Institute for Fisheries Science, Okayama Prefectural Technology Center for Agriculture, Forestry, and Fisheries, Setouchi, Japan

**Keywords:** peptide hormone, *Marsupenaeus japonicus*, ovary, reproduction, transcriptome, vitellogenesis

## Abstract

Crustacean reproduction has been hypothesized to be under complex endocrinological regulation by peptide hormones. To further improve our understanding of the mechanisms underlying this complex regulation, knowledge is needed regarding the hormones not only of the central nervous system (CNS) such as the X-organ/sinus gland (XOSG), brain, and thoracic ganglia, but also the peripheral gonadal tissues. For example, in vertebrates, some gonadal peptide hormones including activin, inhibin, follistatin, and relaxin are known to be involved in the reproductive physiology. Therefore, it is highly likely that some peptide factors from the ovary are serving as the signals among peripheral tissues and central nervous tissues in crustaceans. In this work, we sought to find gonadal peptide hormones and peptide hormone receptors by analyzing the transcriptome of the ovary of the kuruma prawn *Marsupenaeus japonicus*. The generated ovarian transcriptome data led to the identification of five possible peptide hormones, including bursicon-α and -β, the crustacean hyperglycemic hormone (CHH)-like peptide, insulin-like peptide (ILP), and neuroparsin-like peptide (NPLP). Dominant gene expressions for the bursicons were observed in the thoracic ganglia and the ovary, in the CNS for the CHH-like peptide, in the heart for NPLP, and in the ovary for ILP. Since the gene expressions of CHH-like peptide and NPLP were affected by a CHH (*Penaeus japonicus* sinus gland peptide-I) from XOSG, we produced recombinant peptides for CHH-like peptide and NPLP using *Escherichia coli* expression system to examine their possible peripheral regulation. As a result, we found that the recombinant NPLP increased vitellogenin gene expression in incubated ovarian tissue fragments. Moreover, contigs encoding putative receptors for insulin-like androgenic gland factor, insulin, neuroparsin, and neuropeptide Y/F, as well as several contigs encoding orphan G-protein coupled receptors and receptor-type guanylyl cyclases were also identified in the ovarian transcriptome. These results suggest that reproductive physiology in crustaceans is regulated by various gonadal peptide hormones, akin to vertebrates.

## Introduction

As a one of the most important aquaculture target worldwide, the production of penaeid shrimps/prawns has been steadily increased for over the past 30 years. The species occupying the majority of current shrimp aquaculture is Litopenaeus vannamei and Penaeus monodon. Their production has increased ~430% from 1998 to 2008, and 190% from 2008 to 2018, achieving 5.7 million tons (Food and Agriculture Organization of the United Nations; www.fao.org/fishery/topic/16140/en). To enable sustainable penaeid shrimp production in future, efficient seed production technique is required. Information on the endocrine system governing reproductive physiology will help to have the similar efficient seed production technique as in other aquatic animals (1, 2).

A number of studies on endocrinological regulation of basic biological functions have so far focused on the central neurosecretory X-organ/sinus gland complex (XOSG) in the eyestalk among various crustacean species. It has been proved that various peptide hormones produced from the XOSG are regulating growth, metabolism, osmoregulation, and reproduction (3, 4), e.g., red pigment concentrating hormone (5), pigment dispersing hormone (6), crustacean hyperglycemic hormone (CHH) (7), molt-inhibiting hormone (MIH) (8), vitellogenesis- or gonad-inhibiting hormone (VIH/GIH) (9), mandibular organ-inhibiting hormone (MOIH) (10), and crustacean female sex hormone (CFSH) (11). Extensive works based on biological activity-oriented peptide purification and subsequent expansions of homologous cDNA cloning have been contributed to find the above peptide hormones. On the basis of these works, VIH is considered as a main regulator of reproductive process in terms of the inhibition of vitellogenin (VG, a major yolk protein precursor) synthesis. Furthermore, roles of peptide hormones from the central nervous system (CNS) other than XOSG, which includes the brain and thoracic ganglia, have been elucidated (12–14).

In Japan, the principal penaeid species is the kuruma prawn *Marsupenaeus japonicus* (former *Penaeus japonicus*), which is one of the most important aquatic resources. The reproductive processes of *M. japonicus* have also been extensively studied using vitellogenesis-related proteins and their genes (e.g., VG, cathepsin C, cortical rod protein, and thrombospondin) as indices of ovarian development (15–21). Among various peptide hormones which have been purified and characterized from the central XOSG (22–27), six type-I peptides of the crustacean hyperglycemic hormone (CHH) superfamily inhibit the expression of *VG* in the ovary (28, 29). Consequently, the six CHHs, called as *P. japonicus* sinus gland peptide-I (Pej-SGP-I), -II -III, -V, -VI, and –VII, have been hypothesized to be vitellogenesis-inhibiting hormones (VIHs), which explains why eyestalk-ablation acceralates ovarian development. In contrast to the hormones from central XOSG, only a few gender and reproductive organ-specific peripheral factors have been identified. An insulin-like androgenic gland factor of *M. japonicus* (Maj-IAG) is exclusively produced from the androgenic gland of the male gonad and supresses *VG* expression in the ovary (30, 31), which is presumed to control the development of male characteristics, like the orthologs in other decapods (32–34). An ovarian isoform of the crustacean female sex hormone of *M. japonicus* (Maj-CFSH-ov) is dominantly expressed in the ovary, but its function remains to be determined (35). Since some gonadal hormones, activin/inhibin (36–38), follistatin (39), and relaxin (40, 41), are known to be involved in the regulation of reproductive physiology in vertebrates, more attention should be paid for peptidergic factors from the gonad as well as the other peripheral tissues. Such factors may act as a feedback signal from the ovary to the CNS or as a signal that intermediate two VG synthetic site, the hepatopancreas and ovary, in female penaeid shrimps (42).

More recently, transcriptomic analysis has become an important tool for peptide/protein profiling in addition to the conventional approaches described above. The transcriptome data supports our comprehensive understanding of the mechanisms where target tissues are regulated. Indeed, many more transcripts encoding hormones homologous to those found in vertebrates or insects have been identified in various crustacean species (43–52). Some of the studies have shown the existence of transcripts for putative peptide hormones in the ovary: the red swamp crayfish *Procambarus clarkii* (47), the Australian red-craw crayfish *Cherax quadricarinatus* (49), and the giant freshwater prawn *Macrobrachium rosenbergii* (51). In addition to the peptide/protein hormone candidates, the transcriptomic data have been exploited for the identification and characterization of peptide hormone receptors (53, 54) and the elucidation of molecular pathways involved in the regulation of various biological functions (55, 56). Hence, transcriptomics is an essential tool in the quest to improve our understanding of reproductive biology underlying peptide hormones and their receptors in peripheral organs such as the gonads.

Herein, we performed next-generation RNA sequencing on *M. japonicus* ovary with the Illumina MiSeq system. The *de novo* assembled ovarian transcriptome data was searched for the putative peptide hormone precursors and peptide hormone receptors. Moreover, the effects of putative hormones on ovarian *VG* expression were examined using their recombinant peptides.

## Materials and Methods

### Total RNA Extraction

Adult *M. japonicus* were purchased from a local fish market in Okayama Prefecture, Japan. For tissue-specific gene expression analysis, the brain, eyestalk, thoracic ganglia, heart, hepatopancreas, intestine, and gonad were dissected from both three male (20.7 g average body weitht) and three female prawns (23.0 g average body weight; 1.0% average gonadosomatic index, GSI). The prawns are determined to be in intermolt (C0–C1) and early premolt (D0) stages thorugh the observation of the setal development of the pleopods using a method modified from the previous report (57). Ovarian developmental stages of the female prawns were determined as previtellogenic by histlogical analysis (20, 35). Tissues were stored in RNA*later* solution (Thermo Fisher Scientific, MA, USA) at −20°C until further use. For RNA-sequencing, the ovary of two intermolt female prawns in previtellogenic stage (26.4 g body weight; 1.0% GSI) and early exogenous vitellogenic stage (56.3 g body weight; 2.5% GSI) were dissected out and stored as described above. Total RNA was isolated using the illustra RNAspin mini RNA isolation kit (GE Healthcare Bio-Sciences AB, Uppsala, Sweden).

### Library Preparation and RNA-Sequencing

The concentration of the two total RNA samples isolated from the ovary was measured using the Qubit RNA BR assay kit (Thermo Fisher Scientific). The cDNA libraries were constructed with 1 μg of the total RNA using the NEBNext ultra directional RNA library prep kit for Illumina, NEBNext Poly(A) mRNA Magnetic Isolation Module, and NEBNext multiplex oligos for Illumina (index primers set 1; New England BioLabs, MA, USA). All protocols were performed according to the manufacturer's instructions with minor modifications, namely that the fragmentation of RNA was performed by 94°C followed by incubation for 7.5 min. The final library fragment size was estimated to be 200–870 bp (average of 520 bp) using the Agilent high sensitivity DNA kit (Agilent Technologies, CA, USA). The library was sequenced using the MiSeq with Reagent Kit v3 (Illumina, CA, USA) in the paired-end mode with a read length of 300 bases.

### Data Processing and Bioinformatic Analyses

Bases with a quality score (QV < 20) were trimmed from the 5′ and 3′ ends of each read, and reads containing ≥ 30% of low quality bases (QV < 14) with <25 bp were removed before assembling. The preprocessed reads were assembled using the Trinity platform (58). The resultant contigs were analyzed using the Basic Local Alignment Search Tool + (BLAST+; version 2.3.0) to perform a homology search against the National Center for Biotechnology Information non-redundant (NCBI-nr) protein database (ver. 170504) with an E-value cutoff of 1 × 10^−5^. The above-mentioned operations were executed using the DNA Data Bank of Japan (DDBJ) Read Annotation Pipeline and the supercomputer at the Research Organization of Information and Systems (ROIS), National Institute of Genetics (NIG), Japan. The BLAST output contained a maximum of 30 hits for each sequence which were used to assign the functional Gene Ontology (GO) terms to the protein sequences and for further GO Slim analysis on Blast2GO (59, 60). The signal peptide was predicted using SignalP 4.1 Server (61).

### Molecular Cloning of Open Reading Frames of Hormonal Genes

Poly (A)+ RNA was prepared from 50 μg of the total RNA as described above using the NEBNext Poly(A) mRNA Magnetic Isolation Module (New England BioLabs). First-strand cDNA was synthesized from the purified Poly(A)+ RNA using the Transcriptor First Strand cDNA Synthesis kit (Roche Diagnostics, Mannheim, Germany) and an anchored-oligo(dT)_18_ primer. This first-strand cDNA was purified with AMPure XP (Beckman Coulter, IN, USA) and tailed with poly(A) using terminal transferase (Roche Diagnostics). The following PCRs were performed to obtain the correct open reading frame (ORF) of each target gene using the synthesized cDNA.

For 5′ -RACE, PrimeSTAR HS DNA polymerase or TaKaRa LA Taq DNA polymerase (Takara Bio, Shiga, Japan) were used with the cDNA on conventional PCR programs as per a previously described method (35). Adapter1 and adapter2, as forward primers, and bursA-R01 and -R02, as reverse primers, were used for the bursicon A subunit. Adapter1, adapter2, bursA-R01, and -R02 were used for the bursicon B subunit. Adapter1, adapter2, ilp-R01, -R07, -R10, R17, and R18 were used for the insulin-like peptide (Supplementary Table 1).

All PCR products were subcloned into the pGEM-T easy vector (Promega, WI, USA) after the addition of an adenine nucleotide at the 3′ ends. All plasmids were then sequenced on the 3730xl DNA analyzer (Applied Biosystems, CA, USA).

### Construction of Plasmids for Recombinant Peptides

Expression plasmids for the *M. japonicus* CHH-like peptide and *M. japonicus* neuroparsin-like peptide (Maj-pCHH-B and Maj-NPLP, respectively), were prepared as per a previously described method (62). Both Maj-pCHH-B and Maj-NPLP cDNA fragments were amplified by PCR using the pchhbexF1/R1 and nplexF/R primer pairs, respectively (Supplementary Table 1). Each PCR product was mixed with the pET-44a(+) plasmid (Novagen, WI, USA), digested using *Sma* I and *Eco*R I (New England BioLabs), purified with AMPure XP, and then ligated. The thrombin protease cleavage site of the recombinant Maj-pCHH-B (rMaj-pCHH-B) expression plasmid was modified to a tobacco etch virus (TEV) protease cleavage site using the pchhbexF2/R2 primer pair (Supplementary Figure 1). Additionally, the thrombin protease cleavage site of the recombinant Maj-NPLP (rMaj-NPLP) expression plasmid was modified to a human rhinovirus 3C (HRV 3C) protease cleavage site using the nplexF2/R2 primer pair as per a previously described method (62). These modifications accompanied the substitution of N-terminal residues from Gln to Gly in rMaj-pCHH-B and from Ala to Gly in rMaj-NPLP (Supplementary Figure 2).

### Expression and Purification of rMaj-pCHH-B and rMaj -NPLP

The transformation and culture of *E. coli* strain BL21(DE3) STAR (Thermo Fisher Scientific) with expression plasmids, induction of recombinant protein overexpression, and preparation of the soluble fraction of the cell lysate were performed as per previously described methods (62, 63).

The soluble fraction of the cell lysate containing the recombinant fusion protein, His-Nus-His-tagged rMaj-pCHH-B, was purified using the Ni Sepharose 6 Fast Flow resin (GE Healthcare). While the recombinant fusion protein was captured with the resin, the affinity tags were cleaved from rMaj-pCHH-B by protease digestion in a buffer containing 25 mM Tris-HCl (pH 7.4), 0.1 mM EDTA, 0.4 M urea, and ProTEV protease (Promega) at 20°C for 24 h. The untagged rMaj-pCHH-B was washed out from the resin and further purified by reverse phase high-performance liquid chromatography (RP-HPLC) on a Capcell Pak C18 SG300 column (150 × 6 mm; Shiseido, Tokyo, Japan) using the following program: a 2-min hold at 5% acetonitrile (MeCN) in 0.05% trifluoroacetic acid (TFA), a 5-min linear gradient of 5–25% MeCN in 0.05% TFA, a 15-min gradient of 29– 37% MeCN in 0.05% TFA, a 1.2-min gradient of 37– 85% MeCN in 0.05% TFA, and a 5-min hold at 85% MeCN in 0.05% TFA at a flow rate of 0.8 mL/min.

The soluble fraction containing recombinant His-Nus-His-tagged rMaj-NPLP was incubated with the Ni Sepharose 6 resin at 4°C for 20 h. The resin was then washed with washing buffer (20 mM phosphate buffer, 0.2 M NaCl, 50 mM imidazole, pH 7.4) and equilibrated with washing buffer without imidazole. For the cleavage of the tags, HRV 3C protease (Takara Bio) was added, and the resin slurry was incubated at 4°C for 3 days. Untagged rMaj-NPLP was eluted from the resin and further purified by RP-HPLC on the aforementioned column using the following program: a 1-min hold at 5% MeCN in 0.05% TFA, a 4-min linear gradient of 5–21% MeCN in 0.05% TFA, a 15-min gradient of 21– 29% MeCN in 0.05% TFA, a 3.25-min gradient of 29–85% MeCN in 0.05% TFA, and a 5-min hold at 85% MeCN in 0.05% TFA at a flow rate of 0.8 mL/min.

The mass spectra of the purified recombinant peptides were measured on an Agilent 6,520 Accurate-Mass Quadrupole-TOF mass spectrometer with an electrospray ionization (ESI) interface (Agilent Technologies) as we have previously described (62).

### *Ex-vivo* Ovarian Incubation

The effect of one of the CHH family of peptides (Pej-SGP-I) on the mRNA expression of the putative hormones was assessed using our *ex-vivo* ovarian incubation system (28, 29). The same system was also used to assess the effects of rMaj-pCHH-B and rMaj-NPLP on *vitellogenin* (*Maj-VG*) expression. The recombinant Pej-SGP-I (rPej-SGP-I) was prepared as per previously described methods (62–64). Adult female prawns (22.5 g average body weight; 0.9% average gonadosomatic index, GSI) acted as donors of the ovary. As shown in Supplementary Figure 3, the abdominal part of the ovary, where left and right ovarian lobes were sticking to each other, was dissected out and divided into two lobes, and one lobe was incubated as control lobe (medium only), whereas the other received the hormone treatment (experimental). The adjacent part of ovary was kept as initial sample (without incubation) and as sample for histological analysis to determine the vitellogenic stage. Only a single sample set of the ovary (initial, control, and experimental) was prepared from one prawn and counted as *n* = 1; total 30 prawns were used for the experiment in **Figure 5** (6 for each graph), 28 for **Figure 6A**, 24 for **Figure 6B**, and 12 for **Figure 6C**. All prawns used had immature ovary and were in intermolt to early premolt (C0, C1, D0, and D1) stages.

Following incubation, the ovarian tissue fragments were immersed in RNA*later* solution and stored at −20°C. Total RNA extraction was then performed as described above.

### Quantitative Real-Time Reverse Transcriptase PCR

Quantitative real-time reverse transcriptase PCR (qRT-PCR) was used for the quantification of the putative hormone genes, *Maj-VG*, and *arginine kinase* (*Maj-AK*). The sequences of primers and TaqMan probes used in this study have been listed in Supplementary Table 2. The concentrations of total RNA prepared from various tissues were quantified using the Qubit RNA BR assay kit, and for each sample 8 ng RNA was used for qRT-PCR. The qRT-PCR reactions were carried out using the iTaq universal probes one-step kit (Bio-Rad, CA, USA), and the same methods were used for the real-time monitoring of the fluorescence signal on the CFX96 real-time PCR detection system (Bio-Rad) as has been previously described (35). For the quantification of gene expression levels, relative standard curve method was used. DNA templates were amplified with respective gene-specific primer sets (Supplementary Table 2) so that they include qRT-PCR amplicon sequence of respective target genes. RNA standards were synthesized by *in vitro* transcription using *in vitro* Transcription T7 Kit (Takara Bio) with the DNA templates. The synthesized RNA standards were purified using the illustra RNAspin mini RNA isolation kit and quantified using the Qubit RNA BR assay kit as described above. The standard curves were generated using each RNA standard ranging from 40 ng to 0.4 pg prepared by serial 10-fold dilutions, and arbitrary values ranging from 40,000 to 0.4 were assigned correspondingly. Relative gene expression levels were determined based on the threshold cycles using the standard curves. Each standard had almost the same length (371–426 nt, Supplementary Table 2), and similar amplification efficiencies were achieved (95.9–99.8%) in the qRT-PCR.

For the incubated ovary samples, the relative expression of the target genes were standardized to those of *Maj-AK*, and expressed as a percent change from the initial (**Figure 5**) or 0 nM group (**Figure 6**) samples (35) (Supplementary Figure 3). In contrast, for tissue-specific gene expression analysis (**Figure 4**), the relative expression of the target genes were standardized to the total RNA input in the qRT-PCR to account for variations in the *Maj-AK* expression between tissues.

### Statistical Analysis

Gene expression levels have been represented as the mean ± standard error mean (SEM). Statistical differences in gene expression levels were analyzed using the Wilcoxon signed rank test or one-way analysis of variance (ANOVA) followed by the Dunnett's *post hoc* test in the GraphPad Prism version 4.0 for Windows (GraphPad Software, CA, USA).

## Results

### RNA Sequencing and *de novo* Assembly

*De novo* assembly produced 98,509 contigs with a total size of ~91.8 Mbp. The N50 of the transcriptome was 1,676 bp long, and the longest contig was 15,684 bp long. Redundant contigs were eliminated based on a sequence similarity. Among the remaining 47,026 contigs, 24,033 (51.1%) had a BLAST hit, and 13,839 (29.4%) were assigned at least one GO term (Supplementary File). Following bioinformatics analysis on the ovarian transcriptome, several transcripts encoding putative hormones were identified as described below.

### Bursicon

**Figure 1:**
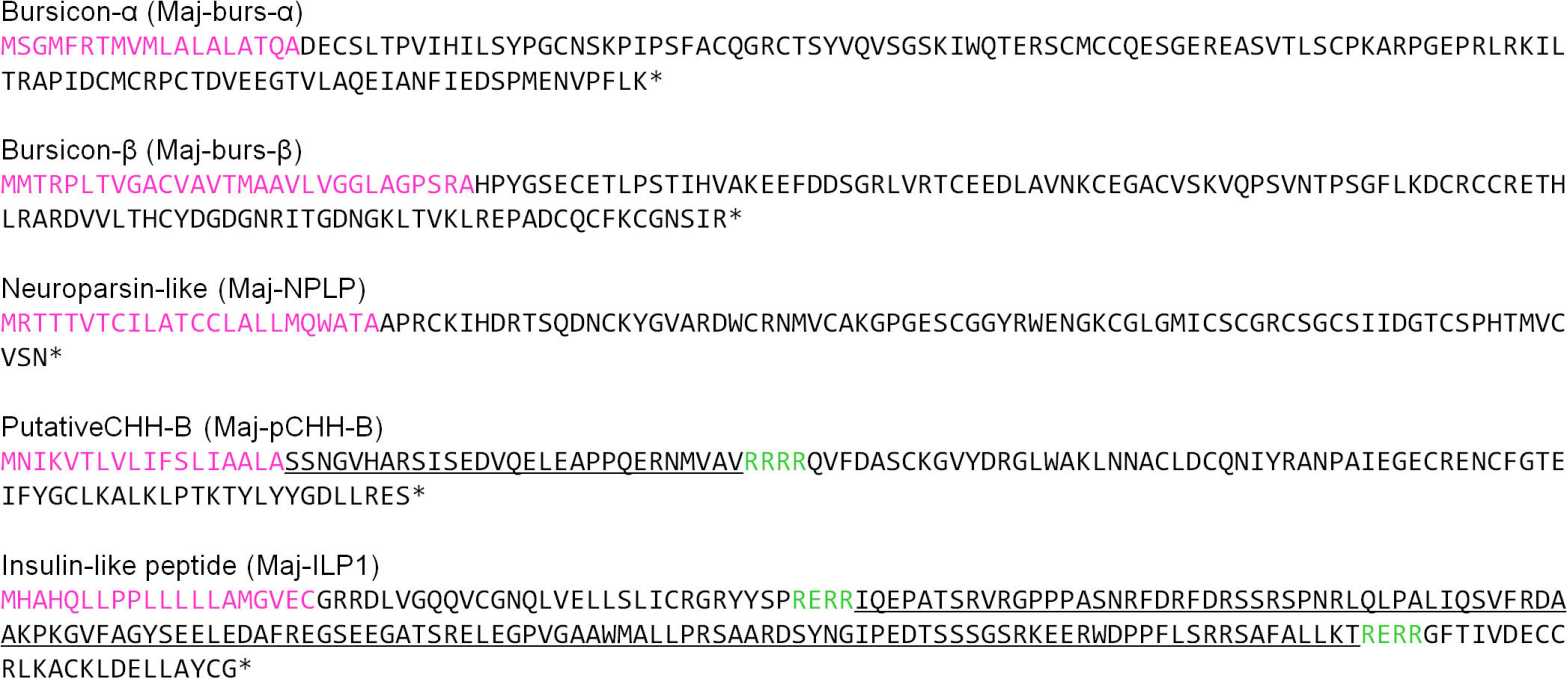
Amino acid sequences of putative hormones. Sequences were deduced from transcriptome analysis of *M. japonicus* ovary and additional cDNA cloning. Sequences shown in magenta and green represent the predicted signal sequences and cleavage sites, respectively. Underlined sequences represent CPRP in Maj-pCHH-B and C-peptide in Maj-ILP1, respectively.

Two contigs putatively encoding bursicon, a heterodimer composed of burs-α and burs–β, were identified in the ovarian transcriptome. Additional cDNA cloning and homology analysis revealed that these were the α and β subunits; Maj-burs-α and Maj-burs-β, respectively. The Maj-burs-α precursor contains a signal peptide (19 amino acid residues) which, once cleaved, gives a mature peptide comprising of 121 amino acid residues. Additionally, the Maj-burs-β precursor also contains a signal peptide (30 amino acid residues) and once processed results in a mature peptide composed of 115 residues (Figure 1). Mature Maj-burs-α and Maj-burs-β both showed ≥ 50% amino acid sequence identity with the known bursicon subunits, and they both contain 11 Cys residues, 9 of which are conserved in the cystine knot-like domain (smart00041 in Conserved Protein Domain Family, NCBI) of the transforming growth factor-β superfamily (Supplementary Figure 4).

### Neuroparsin-Like Peptide

The ovarian transcriptome contained one contig encoding a precursor of Maj-NPLP which contains a 24 amino acid-long signal peptide and 78 amino acid-long mature peptide (Figure 1). We identified 12 conserved Cys residues, which were a characteristic of the crustacean neuroparsin family, in the mature Maj-NPLP peptide. Maj-NPLP shares 46% amino acid sequence identity with an neuroparsin in the greasyback shrimp *Metapenaeus ensis* (MeNPLP) which has been hypothesized to regulate ovarian maturation (65). Additionally, Maj-NPLP shares 32% amino acid sequence identity with the ovary ecdysteroidogenic hormone (OEH) in *Aedes aegypti*, the yellow fever mosquito (66) (Supplementary Figure 5).

### CHH-Family Peptide

**Figure 2:**
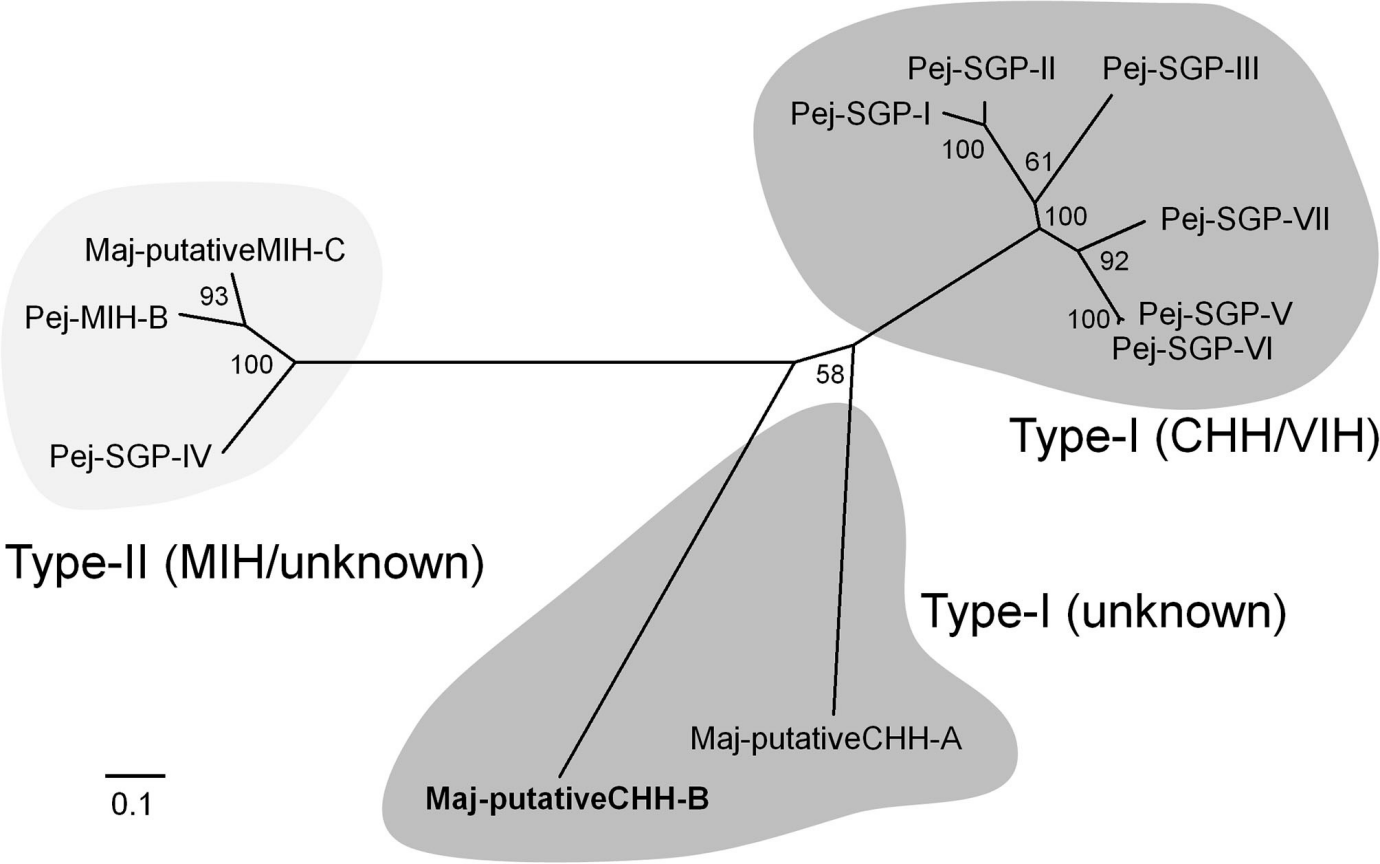
Molecular phylogenetic tree of *M. japonicus* CHH-family peptides. This phylogenetic tree was constructed by the neighbor-joining method with computation of the evolutionary distances using the JTT matrix-based method. Values at the nodes represent the percentage of 1,000 bootstrap replicates. The scale bar shows the number of substitutions per site. Amino acid sequences have been cited from the previous reports (25, 26, 67).

We found one contig encoding the precursor of a CHH-like peptide in the ovarian transcriptome. Additional cDNA cloning revealed that this precursor consisted of a 25 amino acid signal peptide, a 22 amino acid CHH precursor-related peptide (CPRP), an RXRR cleavage signal, and a 74 amino acid mature hormone (Figure 1). Considering the existence of CPRP and the absence of a single Gly residue 5 amino acids downstream of the first Cys residue, we considered this peptide to belong to the type-I subfamily of the CHH superfamily. On the other hand, there was no C-terminal amidation signal, a characteristic feature of type-I subfamily precursors. Additionally, there was a single-residue insertion seven amino acids downstream of the third Cys residue. Based on the primary structure nomenclature of the *M. japonicus* CHH-family peptides (25, 67), we categorized this peptide as a putative CHH-B (Maj-pCHH-B). Its mature peptide sequence showed 37–46% amino acid identity with the *Marsupenaeus* CHH-family peptides (Supplementary Figure 6). Phylogenetic analyses of the CHH superfamily characterized in the XOSG of *M. japonicus* so far (22, 23, 25, 26, 67) suggests that Maj-pCHH-B is diverse and most likely from the typical type-I and type-II subfamilies (Figure 2).

### Insulin-Like Peptide

**Figure 3:**
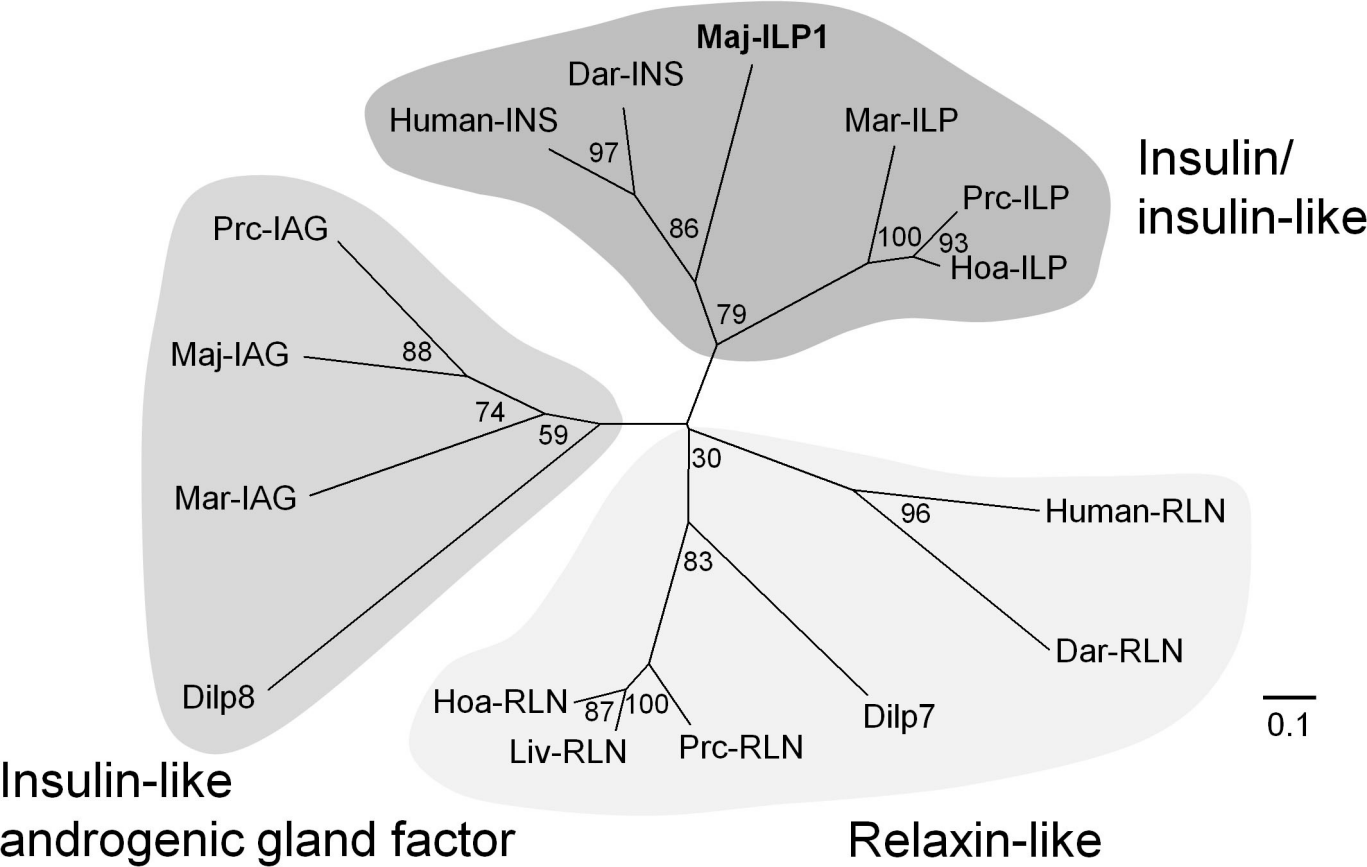
Molecular phylogenetic tree of insulin-family peptides. This phylogenetic tree was constructed by the neighbor-joining method with computation of the evolutionary distances using Poisson correction. Values at the nodes represent the percentage of 1,000 bootstrap replicates. The scale bar shows the number of substitutions per site. Accession numbers of cDNA sequences used are as follows: human-INS, BC005255; human-RLN, A06846; Dar-INS, Dar-RLN, JN215212; Dilp7, NP_570070; Dilp8, NP_648949; Maj-IAG, BAK20460. The other sequences have been cited from a recent report (48).

A putative insulin-like peptide precursor (Maj-ILP1) was identified in the ovarian transcriptome of *M. japonicas*. The full-length ORF of Maj-ILP1 was obtained by several rounds of 5′ -RACE. The precursor comprised a signal peptide composed of 20 residues, a B-chain consisting of 30 amino acid residues, an RXRR cleavage signal, a C-peptide composed of 131 amino acid residues, the other RXRR cleavage signal, and an A-chain composed of 24 amino acid residues (Figure 1). The primary structure of Maj-ILP1 was distinct from IAG in the same species (Maj-IAG); mature peptide (deduced B- and A- chains) of Maj-ILP1 shared only 28.6% amino acid sequence identity with that of Maj-IAG (Supplementary Figure 7). Phylogenetic analysis of known insulin/relaxin family shows that Maj-ILP1 is part of the insulin/insulin-related peptide group and not the IAG or relaxin groups (Figure 3).

### Putative Hormone Receptors

Based on BLAST analysis, several contigs encoding the G-protein coupled receptor family, the receptor guanylyl cyclase family, and the insulin receptor family were found in the present ovarian transcriptome (Table 1). A full-length ORF of contig N13763 was obtained by additional cDNA cloning, and its seven-transmembrane domain showed 62% amino acid sequence identity to the neuropeptide Y (NPY) receptor in the Nevada dampwood termite *Zootermopsis nevadensis*. Additional cDNA cloning also revealed that the ORF of contig N28645 was composed of an N-terminal leucine-rich repeat domain and a seven-transmembrane domain that was highly similar to the transmembrane domain of the relaxin-family peptide receptors (cd15137 in Conserved Protein Domain Family). Furthermore, contigs N06137 and N35023 were the most similar to the receptor for OEH in *A. aegypti* (66) and to IAGR in the Chinese white shrimp *Fenneropenaeus chinensis* (53), respectively.

**Table 1:** Putative hormone receptors found in the ovarian transcriptome.

**Contig name**	**BLAST hit name^**a**^**	**Accession no.^**a**^**	***E*-value^**a**^**
**G-protein coupled receptor homologs**			
N13245	G-protein coupled receptor Mth2-like [*Hyalella azteca*]	XP_018019721	5.5 × 10^−64^
N13763	Substance-P receptor-like, partial [*Limulus polyphemus*]	XP_013785397	1.0 × 10^−94^
N14869	Dopamine D2-like receptor [*Centruroides sculpturatus*]	XP_023239863	5.0 × 10^−105^
N16469	G-protein coupled receptor 143-like [*Frankliniella occidentalis*]	XP_026293244	6.0 × 10^−81^
N21540	Parathyroid hormone-related peptide receptor-like [*Eufriesea mexicana*]	XP_015923473	5.0 × 10^−14^
N28645	G-protein coupled receptor GRL101 [*Sagmariasus verreauxi*]	ARK36624	1.0 × 10^−56^
N38792	Adenosine receptor A2b-like [*Copidosoma floridanum*]	XP_014203447	5.0 × 10^−25^
**Receptor guanylyl cyclase homologs**			
N05354	Guanylate cyclase PcGC-M2 precursor [*Procambarus clarkii*]	AAQ74970	0
N33402	Receptor guanylyl cyclase [*Callinectes sapidus*]	AAX11210	3.0 × 10^−13^
**Insulin receptor homologs**			
N06137	Insulin-like peptide receptor [*Orchesella cincta*]	ODM98443	1.0 × 10^−169^
N15818	Insulin-like receptor [*Cryptotermes secundus*]	PNF35478	3.0 × 10^−68^
N18043	Insulin-like receptor [*Trachymyrmex septentrionalis*]	KYN41515	1.0 × 10^−123^
N35023	Insulin-like androgenic hormone receptor [*Penaeus chinensis*]	AVU05021	1.0 × 10^−88^

a *BLAST hit names, accession numbers, and E-values of some contigs are different from those in Supplementary File because updated database was used for the BLAST search in this table*.

### Tissue-Specific Expression of the Putative Hormones

**Figure 4:**
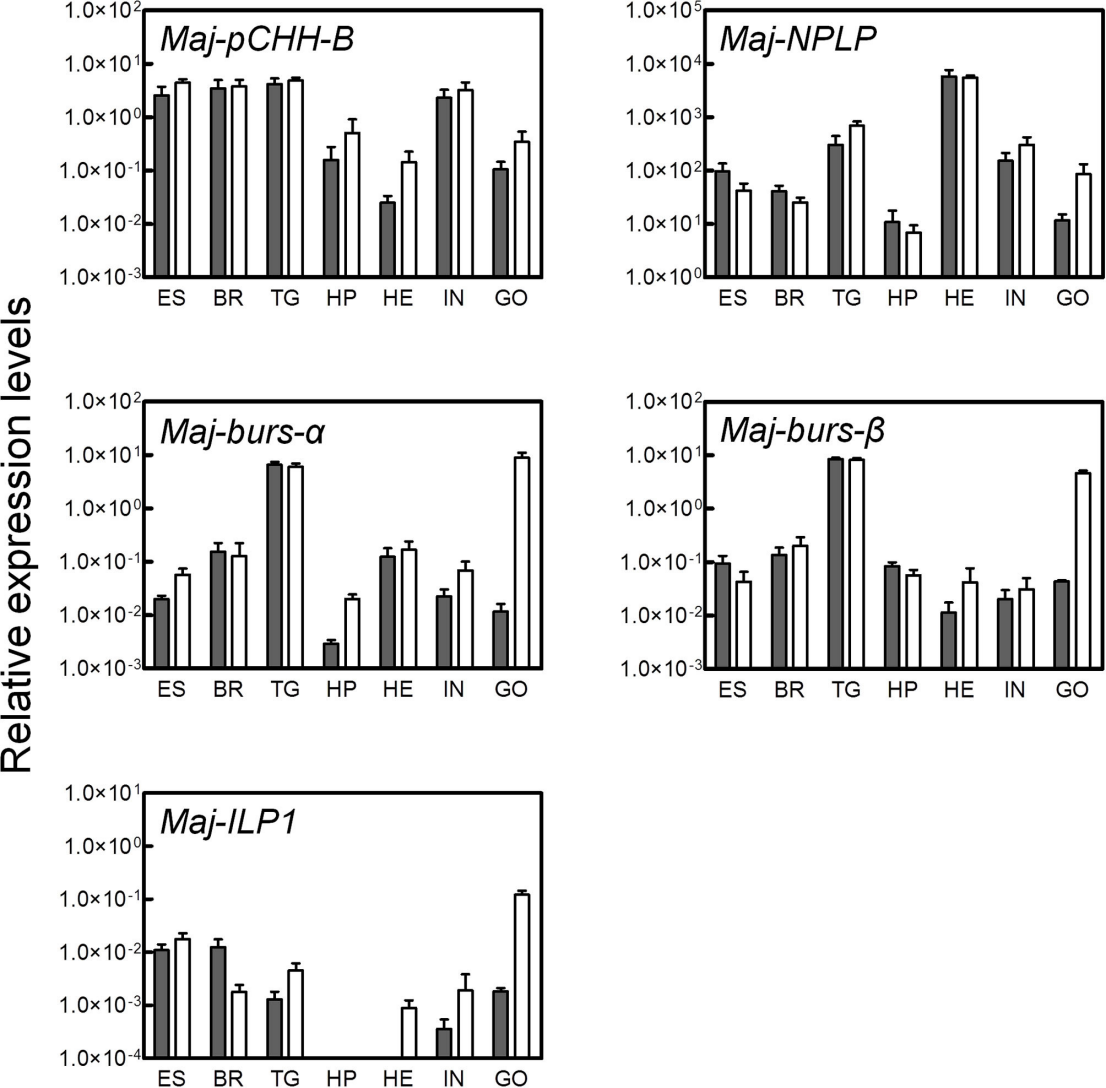
Tissue-specific expression analysis of the putative hormones. Gene expression of the putative hormones were examined by qRT-PCR in various tissues (ES, eyestalk; BR, brain; TG, thoracic ganglia; HP, hepatopancreas; HE, heart; IN, intestine; GO, gonad). Relative expression levels per 8 ng of the total RNA have been represented as mean ± SEM (*n* = 3). Open and solid bars represent female and male prawns, respectively.

Tissue-specific expression levels of the putative hormone genes were examined by qRT-PCR analysis (Figure 4). We found that *Maj-pCHH-B* was mainly expressed in the nervous system and in the intestine of both male and female prawns. Expression in the ovary was low compared to the nervous system, and we did not detect any significant sexual dimorphism in the expression pattern. Additionally, *Maj-NPLP* was expressed primarily in the heart. Apparent expression was also observed in the thoracic ganglia and in the intestine. *Maj-burs-*α and *Maj-burs*-β are primarily expressed in the thoracic ganglia of both sexes and in the ovary. The levels of *Maj-burs-*α and -β expression in the ovary were significantly higher compared to those in the testis. *Maj-ILP1* displayed a gender-specific expression pattern as it was expressed primarily in the ovary. Clear expression was also observed in the nervous system, while no expression was observed in the hepatopancreas of both sexes.

### Effect of Eyestalk VIH on the Expression of the Putative Hormones

**Figure 5:**
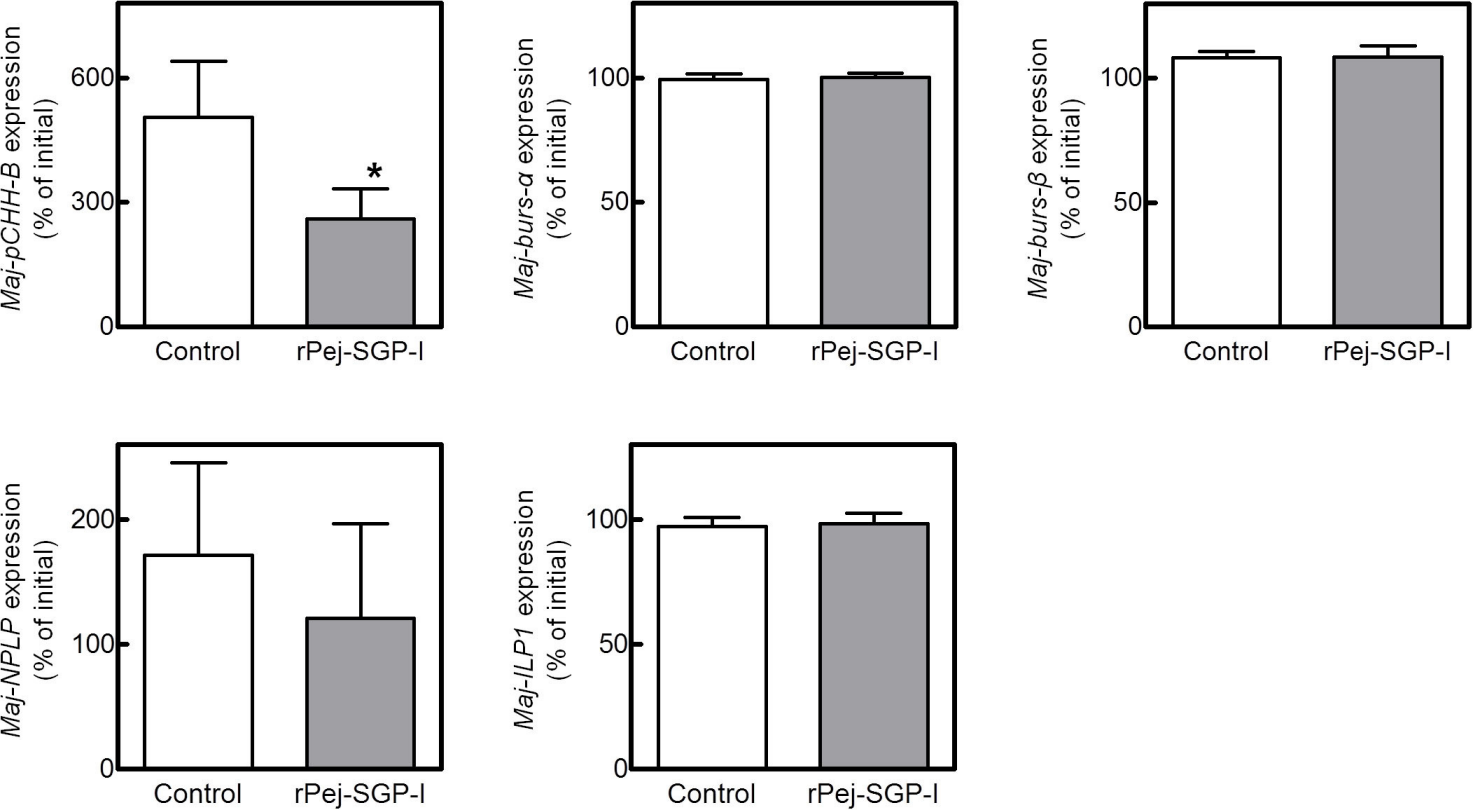
Effect of rPej-SGP-I on mRNA levels of the putative hormones in the ovary. Relative expression levels have been shown as the percentage change relative to that of the initial expression (mean ± SEM, *n* = 6). Statistical differences between control and rPej-SGP-I-treated groups were analyzed by the Wilcoxon signed rank test (*P < 0.05).

To investigate the potential involvement of the putative hormones in vitellogenesis, the effects of Pej-SGP-I, a VIH of *M. japonicus* (28), on hormone gene expression were first examined (Figure 5). In an *ex-vivo* ovarian incubation system, we found that *Maj-pCHH-B* mRNA level was significantly reduced by 50 nM rPej-SGP-I. *Maj-NPLP* expression was also reduced by rPej-SGP-I, but not changed (*p* = 0.063). *Maj-burs-*α, *Maj-burs-*β, and *Maj-ILP1* mRNA levels were not affected by rPej-SGP-I.

### Preparation of rMaj-NPLP and rMaj-pCHH-B

Based on the above results, we further investigated potential involvement of Maj-NPLP and Maj-pCHH-B in vitellogenesis using recombinant peptides. rMaj-NPLP and rMaj-pCHH-B were expressed both as Nus-tagged fusion proteins; they were mostly recovered in the soluble fraction of cell lysates and successfully purified using a Ni-sepharose resin and subsequent HPLC. The deconvoluted mass spectra of untagged and HPLC-purified recombinant peptides have been shown in Supplementary Figure 8. Electrospray ionization (ESI) mass spectrum of rMaj-NPLP revealed a molecular mass of 8,357.1 which agreed with the calculated value (8,368.7) minus 12 Da, suggesting the presence of 6 disulfide bonds in the structure. Similarly, the observed molecular mass of rMaj-pCHH-B was 8,391.7 which was similar to the calculated value (8,397.6) minus 6 Da, indicating the presence of 3 disulfide bonds in the structure.

### Effect of rMaj-NPLP and rMaj-pCHH-B on Ovarian VG Expression

**Figure 6:**
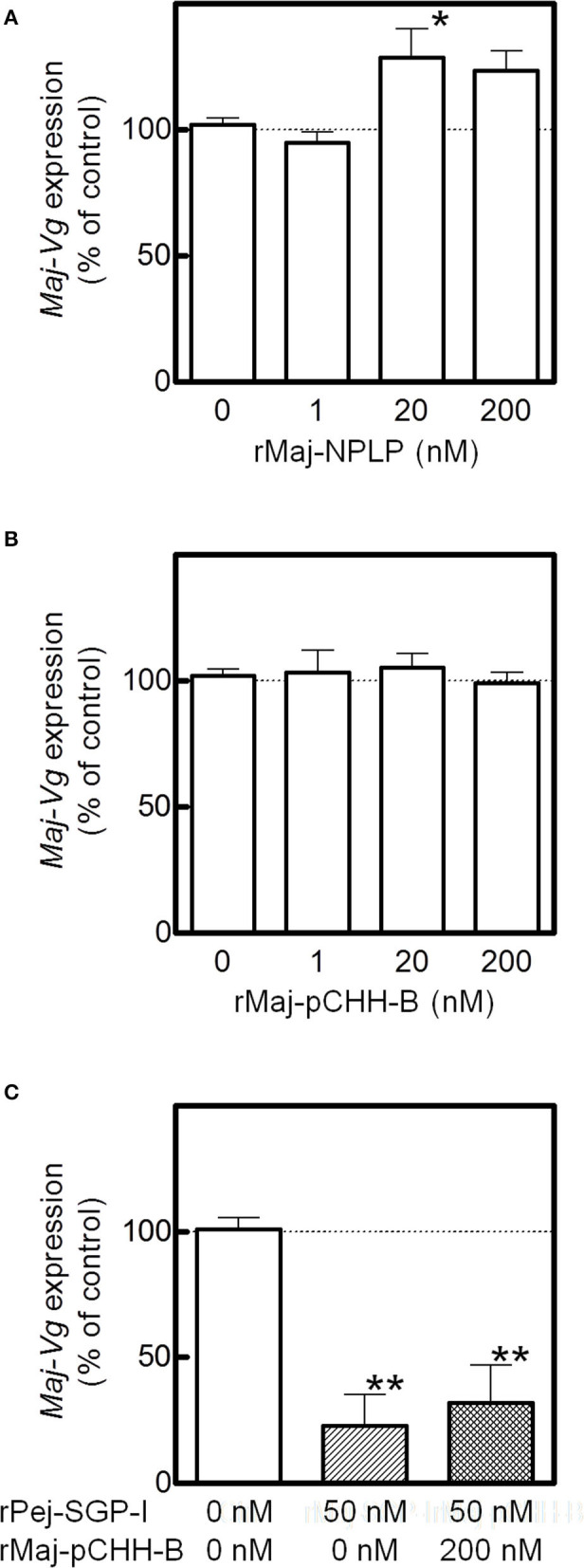
Analyses of rMaj-NPLP and rMaj-pCHH-B functions. *Maj-VG* expressions in *ex-vivo* ovarian fragments incubated with **(A)** rMaj-NPLP and **(B)** rMaj-pCHH-B are examined. Expression levels have been represented as the percentage change relative to those of 0 nM control groups. The differences between controls and the other groups are tested for significance using a one-way ANOVA followed by the Dunnett's post test (*P < 0.05; *n* = 4–8 for rMaj-pCHH-B and 6–8 for rMaj-NPLP). **(C)** *Maj-VG* expression was also examined following incubation without hormones, with rPej-SGP-I alone, and with both rPej-SGP-I and rMaj-pCHH-B. The differences between controls and the other groups are tested for significance using a one-way ANOVA followed by the Dunnett's post test (**P < 0.01; *n* = 4).

The effects of the recombinant peptides on *Maj-VG* expression were assessed in the ovarian incubation system. Although rMaj-NPLP did not affect *Maj-VG* expression at lower doses, *Maj-VG* expression increased at higher doses with significant changes observed in the ovary fragments treated with 20 nM rMaj-NPLP (Figure 6A). In comparison, rMaj-pCHH-B treatment did not affect the *Maj-VG* expression up to 200 nM (Figure 6B). When 200 nM rMaj-pCHH-B was co-incubated with 50 nM rPej-SGP-I, rMaj-pCHH-B acted neither cooperatively nor antagonistically on the vitellogenesis-inhibiting activity of rPej-SGP-I (Figure 6C).

## Discussion

**Figure 7:**
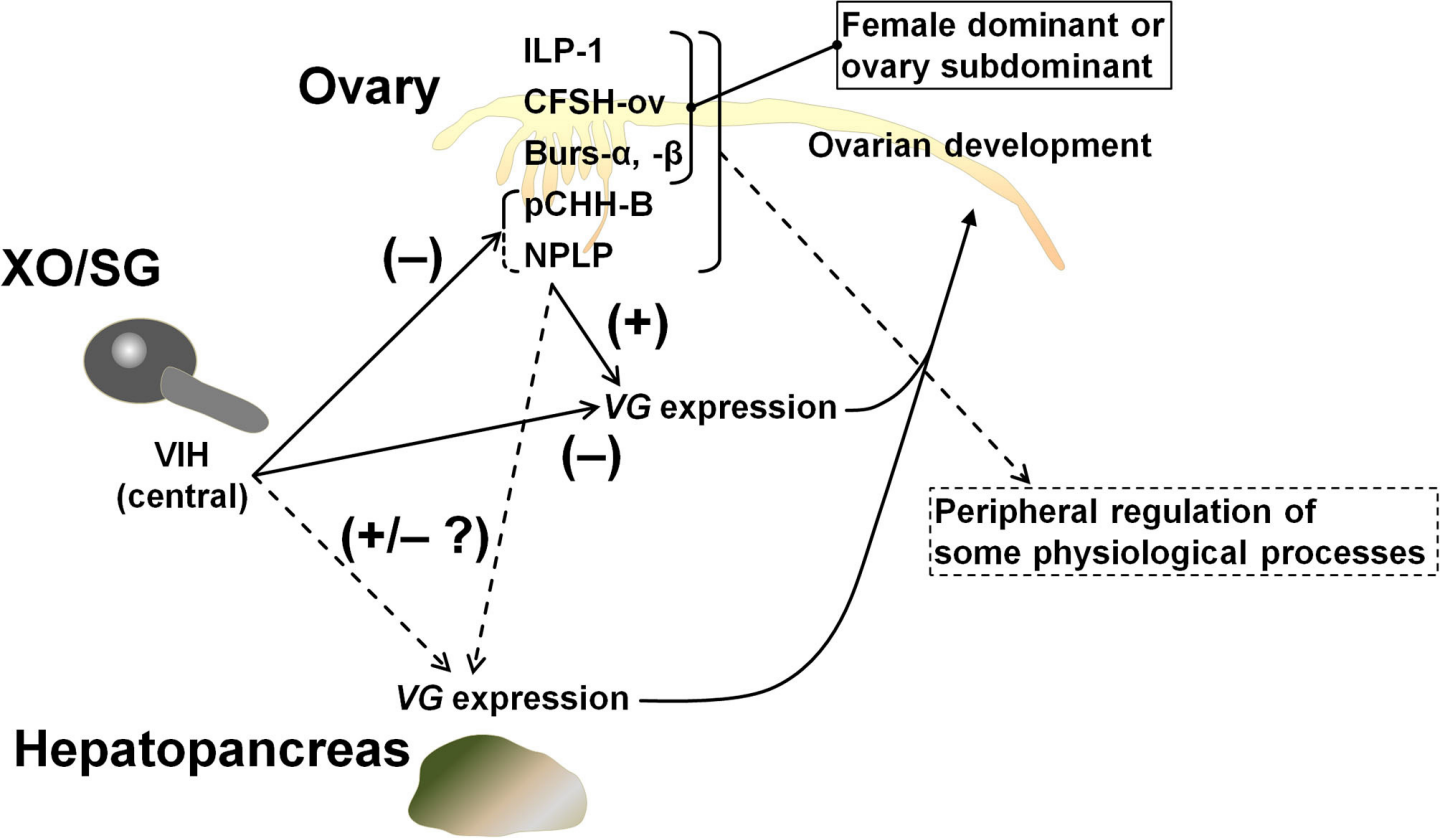
Schematic model of peripheral regulation of the ovary. It is highly likely that Maj-NPLP is a vitellogenesis-regulating factor in *M. japonicus* considering its activity and the existence of a putative receptor in the ovary. The other female dominant or ovary subdominant hormones may play a role in some physiological processes. Maj-CFSH-ov has been previously characterized (35).

The hypothalamus-pituitary-gonad is the main axis controlling vertebrate reproductive processes. In crustaceans, the XOSG and brain ganglia appear to correspond to the axis. Peptide hormones secreted from these niches, especially from the XOSG, have been well-characterized, and their involvements in reproductive processes have been studied. Conversely, there is little information on gonadal hormones. Most penaeid shrimps synthesize VG in the hepatopancreas and in the ovary (42). For example, in *M. japonicus*, the same VG transcript is present in both tissues, but their expression dynamics differ slightly during vitellogenesis (17, 20, 21). Therefore, some peptide factors from the ovary is thought to be serving as feedback signals among peripheral tissues and CNS. Additional studies on gonadal hormones will improve our understanding of the mechanisms behind crustacean reproduction. Thus, in this study, we analyzed the ovarian transcriptome of *M. japonicus* and identified Maj-burs-α, Maj-burs-β, Maj-NPLP, Maj-pCHH-B, and Maj-ILP1 (Figure 1) as some possible peptide hormones produced in *M. japonicus* ovary. Moreover, we examined functions of Maj-NPLP and Maj-pCHH-B. These data, in combination with previous work (35), suggest a peripheral regulation of the ovary as shown in Figure 7 and below.

Regarding neuroparsin, its vitellogenesis-inhibiting activity in terms of the inhibition of the juvenile hormone system has been reported in the migratory locust *Locusta migratoria* (68). In contrast, OEH of the neuroparsin family has a gonadotrophic effect in *A. aegypti* (66, 69); the OEH as well as several ILPs stimulate the ovarian ecdysteroid production, which induces VG synthesis in the fat body. In crustacean species, a neuroparsin-like peptide (MeNPLP) which is produced by the hepatopancreas and has been reported to induce *VG* expression in *M. ensis* (65). Transcriptomic analysis of *Fenneropenaeus merguiensis* (the banana shrimp) ovary shows that the expression of a neuroparsin precursor is higher in the vitellogenic stage compared to that in the non-vitellogenic stage (70). In the present study, we showed that Maj-NPLP has a stimulatory effect on VG synthesis in the ovary (Figure 6A). Although inhibitory effect of Pej-SGP-I on Maj-NPLP expression is not clear (Figure 5), it is likely that other five VIHs (Pej-SGP-II, -III, -V. -VI, and -VII) (28) regulate Maj-NPLP expression in the ovary. Taken together, we concluded that the NPLP is a regulator of reproductive process in arthropods. Multiple isoforms of NPLP are often found in a single crustacean species (47, 49, 65) with differing tissue-specific expression. The expression pattern of *Maj-NPLP* is similar to that of Mar-NP-2 in *M. rosenbergii* (51) which is expressed predominantly in the thoracic ganglia, heart, and gonads. Regarding the Maj-NPLP receptor, contig N06137 (Table 1) has significant sequence similarity to the OEH receptor (66) as well as a similar domain structure (i.e., extracellular venus fly trap domain, a single transmembrane domain, and intracellular tyrosine kinase domain), thereby suggesting that Maj-NPLP may act on the ovary through endocrine or autocrine/paracrine modes of action via this receptor. Such information may be used for further characterization of the role of NPLPs in *M. japonicus* vitellogenesis.

Bursicon, a heterodimer composed of burs-α (burs) and burs-β (pburs), regulates cuticle tanning and wing expansion after ecdysis in insects. Additionally, bursicon of the blue crab *Callinectes sapidus* (CasBurs) appears to be involved in the deposition and thickening of new cuticle as well as granulation of hemocytes (71). Reflecting the constitutive increased expression of the β subunit compared to the α subunit, the ββ homodimer as well as the αβ heterodimer are found in the pericardial organ of *C. sapidus*, but their intrinsic functioning is unknown. However, characteristics of bursicon, a member of the TGF-β superfamily (Supplementary Figure 4) and the dimerization patterns of the subunits, are analogous to those of the activin/inhibin family of proteins which participate in the regulation of reproductive physiology in mammals. Consequently, these data support our hypothesis of the role of bursicon in crustacean reproductive processes. In fact, bursicon has been reported to stimulate *VG* expression in the ovary of *Penaeus monodon*, also known as the black tiger shrimp (13). However, only the heterodimer (i.e., Pmbursα and Pmbursβ subunits) exhibit such a stimulatory effect, whereas the αα and ββ homodimers do not. In contrast, the bursicon receptor ortholog DLGR2, which is encoded in the *rickets* gene in the fruit fly *Drosophila melanogaster* (72), is not found in our *M. japonicus* ovarian transcriptome. Although contig N28645 (Table 1) has a similar domain organization to DLGR2 in terms of the N-terminal leucine-rich repeat domain and the seven-transmembrane receptor domain, their overall sequence similarity is low.

*Maj-pCHH-B* expression was suppressed by a central VIH, suggesting its possible involvement in the regulation of vitellogenesis, but we were unable to show this experimentally using the recombinant peptide (Figure 6B). And Maj-pCHH-B acted neither antagonistically nor cooperatively on the vitellogenesis-inhibiting activity of Pej-SGP-I (Figure 6C). Considering the dominant gene expression pattern in the CNS including the eyestalk, Maj-pCHH-B may act as a neurotransmitter, much like the ion transport peptides (ITP and ITP-L) (73, 74), the ortholog of the CHH superfamily in insects (4, 75). Further functional analysis of Maj-pCHH-B, Mj-putativeCHH, and Mj-putativeMIH-C (25) are required to elucidate the diverse biological functions of the CHH superfamily in *M. japonicus*. As for the primary structure, the single-residue insertion seven amino acids downstream of the third Cys residue (Supplementary Figure 6) is also reported in a CHH from the Pacific white shrimp *Litopenaeus vannamei* (76). Although some venom peptides from spiders and centipedes, which are members of the CHH superfamily, have an unusual number of amino acid residues between the third and fourth or fourth and fifth Cys residues, they share the common tertiary CHH superfamily scaffold (62, 77). Thus, this suggests that Maj-pCHH-B also possess a similar backbone fold.

As shown by our previous studies, six CHH-family peptides from *M. japonicus* XOSG inhibit the ovarian *Maj-VG* expression (28, 29). Hence, the receptor for the CHH-family of peptides is most likely found in the ovary. However, the report of functional CHH-family receptor molecule, in which specific ligand-receptor interaction is proved, is currently very limited (78, 79). Contig N13763 (Table 1) shares 37% amino acid sequence identity with the ITPL receptor in the silkworm *Bombyx mori* (BNGR-A24). Since the ITPL receptor has lower but definite affinity to ITP, N13763 should be investigated as a potential CHH-family receptor in future. In contrast, the contig N13763 shows higher similarity with the NPY receptor. Interestingly, the existence of NPY/F in crustacean species has been reported in some transcriptome analyses (43–49, 51, 52), and NPF has been suggested to stimulate ovarian development in *M. rosenbergii* (80). Therefore, NPF may have a similar function in *M. japonicus*. In *L. vannamei*, it is suggested that a receptor guanylyl cyclase (LvGC) is CHH receptor and is involved in the regulation of *IAG* expression (78). Two contigs encoding receptor guanylyl cyclase family found in the ovarian transcriptome in this study (N05324 and N33402, Table 1) do not contain the extracellular ligand domain, and their sequence similarities cannot be examined.

Recent advances in transcriptomic analysis have revealed multiple molecular species of an insulin family in a single crustacean species. For example, three ILPs (i.e., IAG, ILP, and relaxin-like) have been reported in *L. vannamei, M. rosenbergii*, and *P. clarkii*, respectively (48). Considering the present discovery of Maj-ILP1 and the result of phylogenetic analyses (Figure 3), there may be the third member of the relaxin-like molecular species in *M. japonicus*. Multiple ILPs have also been identified in insects such as *B. mori* (81), *D. melanogaster* (82), and the red flour beetle *Tribolium castaneum* (83). In *T. castaneum* ILP2 and ILP3 regulate *VG* expression in downstream juvenile hormone signaling (84), and in *A. aegypti* ILP3 controls egg production through the stimulation of yolk uptake and ecdysteroid production in the ovary (85). Arthropod ILP has been suggested to be a factor which links nutritional status and reproductive status (86, 87). Taken together, the female-specific and gonad-dominant Maj-ILP1 may also have such functions. Furthermore, our *M. japonicus* ovarian transcriptome contained a putative insulin receptor/IAGR (Table 1). Although *Fenneropenaeus* IAGR is not detected in any female tissues (53), the presence of IAGR homolog in *M. japonicus* ovary can account for the inhibitory effect of Maj-IAG on *Maj-VG* expression (31). The possibly important relationship between the insulin signaling pathway and reproduction should be studied in terms of the ligand as well as the receptor and downstream factors, such as the components of the signaling pathway which have been revealed to be conserved in *M. japonicus* by transcriptome analysis (Supplementary Figure 9).

In summary, we reported putative peptide hormones and receptors obtained through mRNA-sequencing analysis of the ovary of *M. japonicus*. Results of this study suggest a possible peripheral regulation by these hormones in the crustacean reproductive physiology (Figure 7). Factors involved in vitellogenesis regulation, ovary-specific expression patterns, and putative receptors for neuroparsin, ILP, and other peptide hormones are fascinating starting points for further detailed characterization. Above all, the effects of the ovarian hormones as well as central VIH on *Maj-VG* expression in the hepatopancreas should be addressed to outline the endocrine regulation of vitellogenesis. In addition, the transcriptome data generated in this work can also be utilized to further study hormonal functions. For example, target genes with expression patterns that are affected by VIH and other putative hormones can be efficiently searched using the combination with *ex-vivo* culture system and transcriptome analysis. Effective use of transcriptomic data from central and peripheral tissues will allow the comprehensive understanding of the regulatory mechanism of reproduction and other physiological processes in crustaceans.

## Data Availability Statement

The datasets presented in this study can be found in online repositories. The names of the repository/repositories and accession number(s) can be found below: https://www.ddbj.nig.ac.jp/, Data for DRA010103 is available in DDBJ Sequence Read Archive.

## Author Contributions

NT conceived the idea for the project, conducted most of the experiments, and analyzed the results. YK and KI analyzed the transcriptomic data. NT and TS prepared the manuscript. All authors contributed to the article and approved the submitted version.

## Conflict of Interest

The authors declare that the research was conducted in the absence of any commercial or financial relationships that could be construed as a potential conflict of interest.
