# Supplementary material for: Transcriptomic Analysis of the Kuruma Prawn Marsupenaeus japonicus Reveals Possible Peripheral Regulation of the Ovary
Source: Front Endocrinol (Lausanne). 2020 Aug 19;11:541. doi: 10.3389/fendo.2020.00541 (PMC7466434; doi:10.3389/fendo.2020.00541)
Supplement: Supplementary file 1 [file Data_Sheet_1.pdf]

Supplementary Table 1. Primers and used for cDNA cloning and expression plasmid construction

Primer	Sequence
5'-RACE and ORF cloning[a]	
dT-adapter	TATCTAGAGGCCGAGGCGGACGACATG(T)$_{27}$VN
adapter1	TATCTAGAGGCCGAGGCGGAC
adapter2	GGCCGAGGCGGACGACATG
bursA-R01	GCAGGGTCGACACATGCAGTC
bursA-R02	GTCGTGCTTTAGGGCAGCTGAG
bursB-R01	GGATCGAACTCCTTATCGGATGGAG
bursB-R02	CAGTGGGTGAGCACCACGTC
ilp-R01	CACAATACGCCAACAGCTCATCGAG
ilp-R07	AGGAAGTGTCCTCGGGAA
ilp-R10	GCGAAGACGCCCTTGGGCTT
ilp-R17	GGTTCGGGGATCGAGACGA
ilp-R18	AAGCGGTTCGAAGCGGGA
Preparation of recombinant peptides	
pchhbexF1[b]	TTC<u>CCCGGG</u>GTATGCAGGTGTTCGACGCCTCGTG
pchhbexR1[b]	AAA<u>GAATTC</u>ACTAGCTCTCTCTCAAAAGGTCGCCATAG
pchhbexF2	CACCGCCAACTGGTGAGAACCTCTACTTTCAGGGGGTGTTCGAC
pchhbexR2	GTCGAACACCCCCTGAAAGTAGAGGTTCTCACCAGTTGGCGGTG
nplexF1[b]	TT<u>CCCGGG</u>GTCCCAGGTGCAAAAT
nplexR1[b]	AAA<u>GAATTC</u>ATTAATTCGAGACGCAGACCA
nplexF2	TCGCCACCGCCACTTGAAGTGCTCTTCCAGGGTCCCAGGTG
nplexR2	CACCTGGGACCCTGGAAGAGCACTTCAAGTGGCGGTGGCGA

[a]Sequences of primers for anchored-oligo(dT)$_{18}$, adapter1, and adapter2 are described in the previous study (35).

[b]*Sma*I and *Eco*RI restriction sites are underlined, respectively.

Supplementary Table 2. Sequences of primers and TaqMan probes used for qRT-PCR analysis

Primer/probe	Sequence
Quantitative real-time RT-PCR[a]	
bursAq-forward	ACCCCGACTGAGGAAGATTCTC
bursAq-reverse	GGCGAGTCTTCGATGAAGTTG
bursAq-probe	AAGGCACAGTGCTTGCGCAGGAAA
bursBq-forward	GTGCTTCAAGTGCGGAAACTC
bursBq-reverse	AGGTCGCATGGTGAGAATCCT
bursBq-probe	TTCGATCCCTTTCCAACGCTCGTGTT
ilpq-forward	CAGGAGAAGCGCTTTCGCT
ilpq-reverse	CAACAGCTCATCGAGTTTGCA
ilpq-probe	ACCATTGTGGATGAGTGCTGCAGGTTG
nplq-forward	TGCTCCTGTGGCAGGTGTTC
nplq-reverse	TTCGCTCGAGGTCTTAATTCGAG
nplq-probe	ATTGACGGCACATGCTCGCCTCA
pchhbq-forward	GAACTCGAAGCTCCTCCTCAAG
pchhbq-reverse	CCCCTGTCATAGACCCCTTTG
pchhbq-probe	CAACATGGTCGCCGTCCGAAGAA
DNA template preparation for RNA standard[b, c]	
For *Maj-AK* (398 bp)	
STak_F03	CTAATACGACTCACTATAGGGACTCTGGCGTTGGTATCTAT
STak_R02	GAGGAAGTGGTCGTCGATCAA
For *Maj-burs-α* (421 bp)	
STbursA_F01	CTAATACGACTCACTATAGGGACCCCGACTGAGGAAGATTC
STbursA_R02	CTGCGAGAGCGAATACTGTTCTA
For *Maj-burs-β* (426 bp)	
STbursB_F02	CTAATACGACTCACTATAGGGTCAGAATGCGAGACCCTT
STbursB_R01	AGGTCGCATGGTGAGAATCCT
For *Maj-NPLP* (371 bp)	
STnplp_F01	CTAATACGACTCACTATAGGGTGCCGCCATCATGAGAA
STnplp_R02	CTCCTCTCCTTGGCATCTTT
For *Maj-pCHH-B* (419 bp)	
STpchhB_F01	CTAATACGACTCACTATAGGGGAACTCGAAGCTCCTCCTCAA
STpchhB_R02	GAAACTTTCCGATCCGGTTGTT
For *Maj-ILP1* (380 bp)	
STilp1_F02	CTAATACGACTCACTATAGGGCCTTGATTCAGTCCGT
STilp1_R01	CAACAGCTCATCGAGTTTGCA

[a]Sequences of primers and TaqMan probes for *Maj-VG* and *Maj-AK* are described in the previous study (35).
[b]Length of DNA templates for RNA standards are shown in parentheses.
[c]T7 promoter sequences are underlined.
