# Supplementary material for: Transcriptomic Analysis of the Kuruma Prawn Marsupenaeus japonicus Reveals Possible Peripheral Regulation of the Ovary
Source: Front Endocrinol (Lausanne). 2020 Aug 19;11:541. doi: 10.3389/fendo.2020.00541 (PMC7466434; doi:10.3389/fendo.2020.00541)
Supplement: Supplementary file 2 [file Data_Sheet_2.pdf]

Expression vector for Maj-pCHHB

```
cDNA:                        ...CGAAGAAGGCGACAGGTGTTCGACGCCTCGTGCAAAGGGGTC...TACTATGGCGACCTTTTGAGAGAGAGCTAGTGCATGGTC...
AA:                          ...R  R  R  R  Q  V  F  D  A  S  C  K  G  V.....Y  Y  G  D  L  L  R  E  S  *  C  M  V
Primers:                      TTCCCCGGGGTATGCAGGTGTTCGACGCCTCGTG                CTATGGCGACCTTTTGAGAGAGAGCTAGTGAATTCTTT
                                                   pchhbexF1 >                 < pchhbexR1(complementary seq.)

pET44 inserted cDNA:
...GACTCGCCACCGCCAACTGGTCTGGTCCCCCGGGGTATGCAGGTGTTCGACGCCTCGTGCAAAGGGGTC...TACTATGGCGACCTTTTGAGAGAGAGCTAGTGAATTCTGT...
AA: ..S  P  P  P  T  G  L  V  P  R  G  M  Q  V  F  D  A  S  C  K  G  V.....Y  Y  G  D  L  L  R  E  S  *  *
Primers:  CACCGCCAACTGGTGAGAACCTCTACTTTCAGGGGGTGTTCGAC
          < pchhbexR2                       pchhbexF2 >
          (complementary seq.)

pET44 inserted cDNA(modified):
...GACTCGCCACCGCCAACTGGTGAGAACCTCTACTTTCAGGGGGTGTTCGACGCCTCGTGCAAAGGGGTC...TACTATGGCGACCTTTTGAGAGAGAGCTAGTGAATTCTGT...
AA: ..S  P  P  P  T  G  E  N  L  Y  F  Q  G  V  F  D  A  S  C  K  G  V.....Y  Y  G  D  L  L  R  E  S  *  *
                                          <~~~~~~~~~~~~~~~~~~~~~~~~~~~~~~~~~~~~~~~~~~~~~~~~~~~~~~~~~~~~>
                                          rMaj-pCHH-B produced as N-terminally modified mature peptide
```

Supplementary Figure 1. Preparation of the expression vector for Maj-pCHHB.
Primer sequences are also shown in **Supplementary Table 1**. Restriction sites are underlined as in **Supplementary Table 1**. Residues shown in green and Gln residue with circle are predicted as processing site and N-terminus of the mature hormone, respectively (See Fig. 1). Using PCR, the thrombin protease cleavage site (LVPR|GM) is modified to a tobacco etch virus protease cleavage site (ENLYFQ|G); both are shown in rectangular boxes. The modification results in the substitution of N-terminal residue from Gln to Gly (circled) in rMaj-pCHH-B.

Expression vector for Maj-NPLP

```
cDNA:                     ...ACGGCGGCTCCCAGGTGCAAAATTCACGACCGCACG...TGCTCGCCTCACACGATGGTCTGCGTCTCGAATTAA...
AA:                       ...T  A  A  P  R  C  K  I  H  D  R  T.....C  S  P  H  T  M  V  C  V  S  N  *
Primers:                    TTCCCGGGGTCCCAGGTGCAAAAT                                     TGGTCTGCGTCTCGAATTAATGAATTCTTT
                                           nplexF1 >                                     < nplexR1(complementary seq.)

pET44 inserted cDNA:
...GACTCGCCACCGCCAACTGGTCTGGTCCCCCGGGGTCCCAGGTGCAAAATTCACGACCGCACG...TGCTCGCCTCACACGATGGTCTGCGTCTCGAATTAATGAATTCT...
AA: ..S  P  P  P  T  G  L  V  P  R  G  P  R  C  K  I  H  D  R  T.....C  S  P  H  T  M  V  C  V  S  N  *  *
Primers:
      TCGCCACCGCCACTTGAAGTGCTCTTCCAGGGTCCCAGGTG
      < nplexR2                         nplexF2 >
      (complementary seq.)

pET44 inserted cDNA(modified):
...GACTCGCCACCGCCACTTGAAGTGCTCTTCCAGGGTCCCAGGTGCAAAATTCACGACCGCACG...TGCTCGCCTCACACGATGGTCTGCGTCTCGAATTAATGAATTCT...
AA: ..S  P  P  P  L  E  V  L  F  Q  G  P  R  C  K  I  H  D  R  T.....C  S  P  H  T  M  V  C  V  S  N  *  *
                                    <~~~~~~~~~~~~~~~~~~~~~~~~~~~~~~~~~~~~~~~~~~~~~~~~~~~~~~~~~~~~~~~~~>
                                        rMaj-NPLP produced as N-terminally modified mature peptide
```

Supplementary Figure 2. Preparation of the expression vector for Maj-NPLP.
Primer sequences are also shown in **Supplementary Table 1**. Restriction sites are underlined as in **Supplementary Table 1**. Residues shown in magenta and Ala residue with circle are predicted as signal sequence and N-terminus of the mature hormone, respectively (See Fig. 1). Using PCR, the thrombin protease cleavage site (LVPR|GP) is modified to the human rhinovirus 3C protease cleavage site (EVLFQ|GP); both are shown in rectangular boxes. The modification results in the substitution of N-terminal residue from Ala to Gly (circled) in rMaj-NPLP.

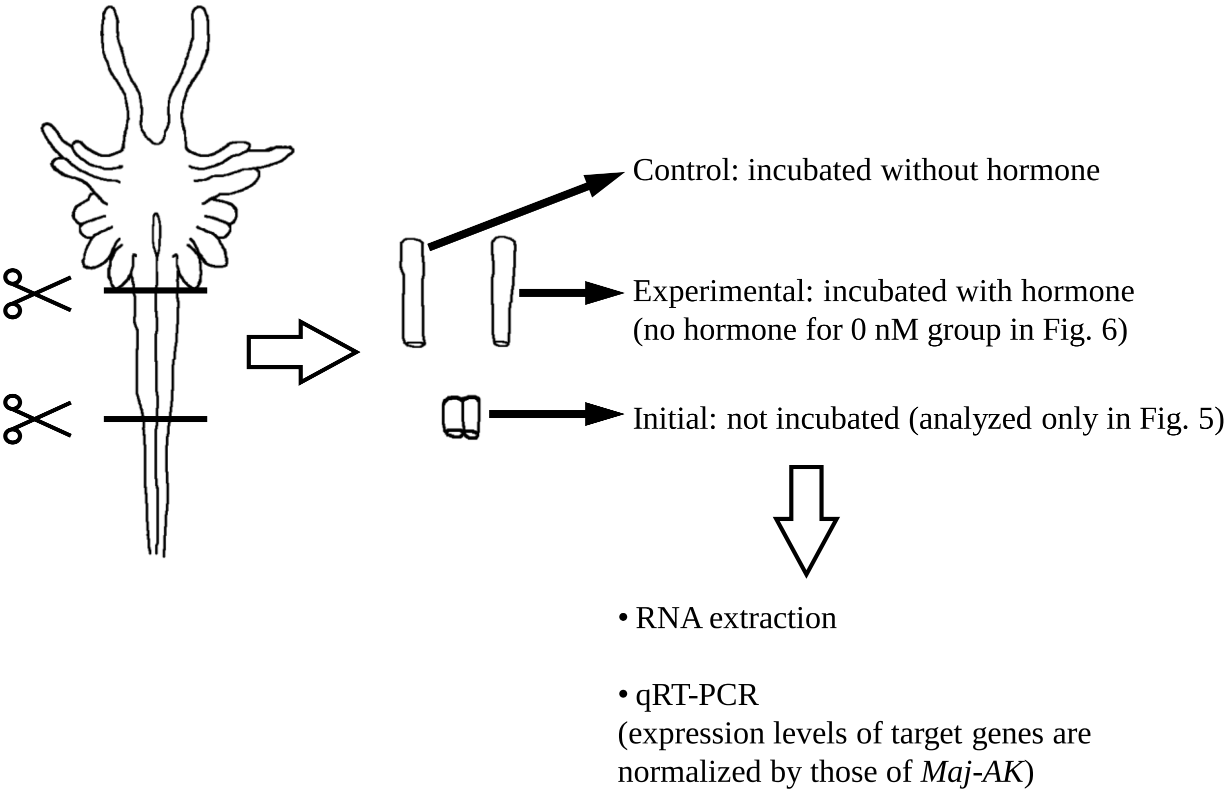

In Fig. 5, expression levels of hormone genes are shown as % of initial.

$$\text{Control: } \frac{\text{Normalized expression level (control)}}{\text{Normalized expression level (initial)}} \times 100$$

$$\text{rPej-SGP-I: } \frac{\text{Normalized expression level (experimental)}}{\text{Normalized expression level (initial)}} \times 100$$

In Fig. 6, *Maj-Vg* expression levels are shown as % of control.

$$\text{0 – 200 nM: } \frac{\text{Normalized expression level (experimental)}}{\text{Normalized expression level (control)}} \times 100$$

Supplementary Figure 3. *Ex-vivo* ovarian incubation.
For details, see Materials and methods and previous reports (28,29,31,35,62).

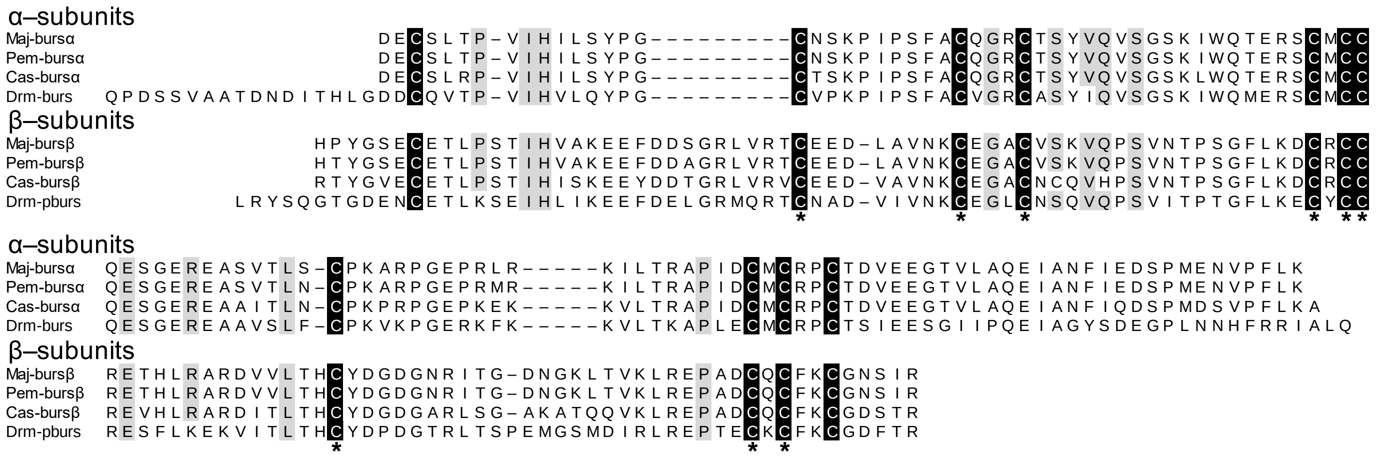

Supplementary Figure 4. Multiple amino acid sequence alignment of bursicons. Amino acid sequences of putative mature peptides have been shown. Conserved Cys residues have been represented as white letters on black background, and those for cystine knot-like domain (smart00041) of the transforming growth factor-β superfamily have been shown as asterisks. Residues conserved in seven or more molecular species have been shown in the grey background. Accession numbers of cDNA sequences used are as follows: Cas-bursα, EU677191; Cas-bursβ, EU677190; Drm-burs, NM_142726; Drm-pburs, NM_135868, Pem-bursα, KP191597; Pem-bursβ, KP191598.

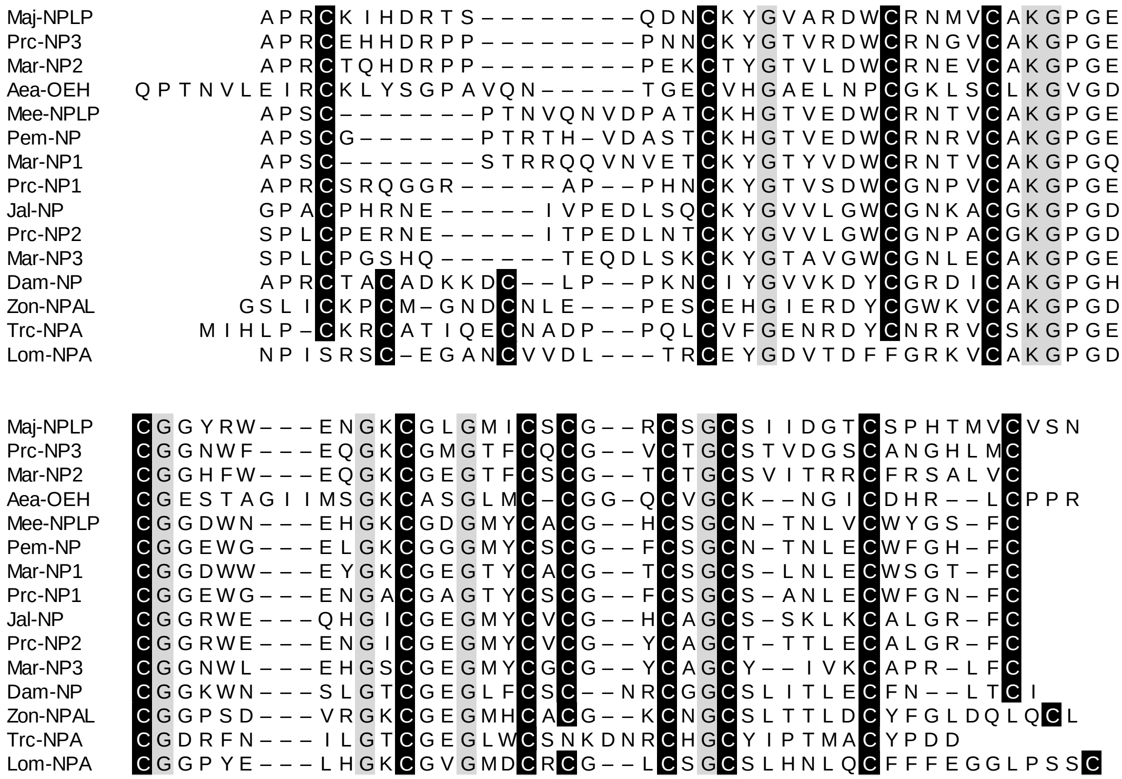

Supplementary Figure 5. Multiple amino acid sequence alignment of neuroparsin and related peptides.

Amino acid sequences of putative mature peptides have been shown. Cys residues have been represented as white letters on black background. The other conserved residues have been shown in the grey background. Accession numbers of cDNA sequences used are as follows: Aea-OEH, AAC38958; Dam-NP, KZS22053; Jal-NP, KF774300; Lom-NPA, P10776; Mee-NPLP, KJ415256; Pem-NP, KT072705; Trc-NPA, XP_008193973; Zon-NPAL, XM_022067413. Amino acid sequences of Mar-NP1 to 3 and Prc-NP1 to 3 are cited from (48).

```
Pej-SGP-I          S L F D P S C T G V F – D R Q L L R R L G R V C D D C F N V F R E – P N V A T E
Pej-SGP-II         S L F D P S C T G V F – D R Q L L R R L G R V C D D C F N V F R E – P N V A M E
Pej-SGP-III        S L F D P A C T G I Y – D R Q L L R K L G R L C D D C Y N V F R E – P K V A T G
Pej-SGP-V          L V F D P S C A G V Y – D R V L L G K L N R L C D D C Y N V F R E – P N V A T E
Pej-SGP-VI         L V F D P S C A G V Y – D R V L L G K L N R L C D D C Y N V F R E – P N V A T E
Pej-SGP-VII        A A F D P S C T G V Y – D R E L L G R L S R L C D D C Y N V F R E – P K V A M E
Pej-SGP-IV (MIH)   S F I D N T C R G V M G N R D I Y K K V V R V C E D C T N I F R L – P G L D G M
Pej-MIH-B          N I L Y S S C R G V M G N R D I Y S K V E R V C N D C T N L Y R L – P Q L D G L
Maj-putativeMIH-C  S I L D S N C R G A M G N R D I Y T K V E R V C E D C T N L Y R L – P Q L D G L
Maj-putativeCHH    D T F D H S C K G I Y – N R Q L F K D L A R V C E D C Y N L Y R K – P Y V A T E
Maj-putativeCHH-B  Q V F D A S C K G V Y – D R G L W A K L N N A C L D C Q N I Y R A N P A I E G E

Pej-SGP-I          C R S N C Y N N P V F R Q C M A Y V V P A H L H N E H R E A V Q M V (amide)
Pej-SGP-II         C R S N C Y N N P V F R Q C M E Y L L P A H L H D E Y R L A V Q M V (amide)
Pej-SGP-III        C R S N C Y H N L I F L D C L E Y L I P S H L Q E E H M A A M Q T V (amide)
Pej-SGP-V          C R S N C F Y N L A F V Q C L E Y L M P P S L H E E Y Q A N V Q M V (amide)
Pej-SGP-VI         C R S N C F Y N L A F V Q C L E Y L L P P S L H E E Y Q A N V Q M V (amide)
Pej-SGP-VII        C R S N C F F N P A F V Q C L E Y L I P A E L H E E Y Q A L V Q T V (amide)
Pej-SGP-IV (MIH)   C R N R C F Y N E W F L I C L K A A N R E D E I E K F R V W I S I L N A G Q
Pej-MIH-B          C R N R C F N N Q W F L L C L N S A K R E D E L N N F R L W I S I L N A G R E W
Maj-putativeMIH-C  C R N R C F N N Q W F L L C L K A T E R Q D E L E N F R L W I S I L N A G R A W
Maj-putativeCHH    C K N N C F V N P K F G H C V A S L N L N V K R Y T K M A H F L R Y S
Maj-putativeCHH-B  C R E N C F G T E I F Y G C L K A L K L P T K T Y L Y Y G D L L R E S
```

Supplementary Figure 6. Multiple amino acid sequence alignment and molecular phylogenetic tree of the *M. japonicus* CHH family of peptides.

Amino acid sequences of mature peptides have been shown. Cys residues have been represented as white letters on black background. Residues conserved in ten or more molecular species have been shown as the grey background.

B-chain and putative cleavage signal

```
Maj-ILP1                     GRRDLVGQQV-CGNQLVELLSLICRGRYYSP[RERR]
Prc-ILP             SQDTYTTSHPEGEPGRRL-CGWRLANKLNRVCKGVYNNPRSTNNYLYY[RGRR]
Mar-ILP              QVSSSDLGEEGKPLRRL-CGWRLANKLNQVCKGIYNKPTVTNNDLFY[RSVR]
Hoa-ILP             SRRTYPTS--EEEPRRRL-CGWRLANKLNLVCKGVYNNPGSTGNYLFY[RSRR]
Maj-IAG                      YNVSGIPVDFDCG-DIGDTMSLICKTF----PTARPYS-[RVSR]
Prc-IAG                      YWVDNLLVDFDCG-NLADTMDSICLTFNEYNDTHLHYAA[RAVR]
Mar-IAG                      YEIECLSVDFDCG-DITNTLASVCLRHNNYINPGPTYVS[KERR]
Prc-RLN  LDPDFVRQIESRTELEWQALWSEERLAL-CRAKLRQNLDAICGKDVF[RR]
Liv-RLN  LDPDLIRQIESRTEAEWQTLWSKERLAL-CRARLRYNLDSICGKDVY[RR]
Hoa-RLN  LEPYLIRQLQSRTEAEWEVLWNKERLAL-CRARLRHNLEAICGKDVY[RR]
```

A-chain

```
Maj-ILP          GFTIVDECCRLKA---CKLDELLAYCG
Prc-ILP              GLSAECCQ-KV---CTVSELVGYCY
Mar-ILP              GLSAECCR-KA---CRVSELMGYCQ
Hoa-ILP              GLSAECCR-KV---CTVSELVGYCY
Maj-IAG          SYNVHDECCNHVSQRMCVAEEILEYCQDPVP
Prc-IAG  QANTDNHVRFNIQDECCNYMRPRTCVLEEITEYCVEPEDGALLTW
Mar-IAG       DSVRRSPREECCNNASFRRCNFEEVAEYCIELRPGVNTCSSR
Prc-RLN         QSPSITTECCTVAG---CTWEEYAEYCPSSNRAR----FL
Liv-RLN         QSPSITAECCTAVG---CTWEEYAEYCPTSSRLRAGVTLI
Hoa-RLN         QSSSITAECCTTVG---CTWEEYAEYCPTSSRLRPGVTPI
```

Supplementary Figure 7. Multiple amino acid sequence alignment and molecular phylogenetic tree of insulin family peptides.

Amino acid sequences of putative B- and A-chains have been shown. Putative cleavage sites have been boxed. Cys residues have been represented as white letters on black background. The other conserved residues have been shown in the grey background.

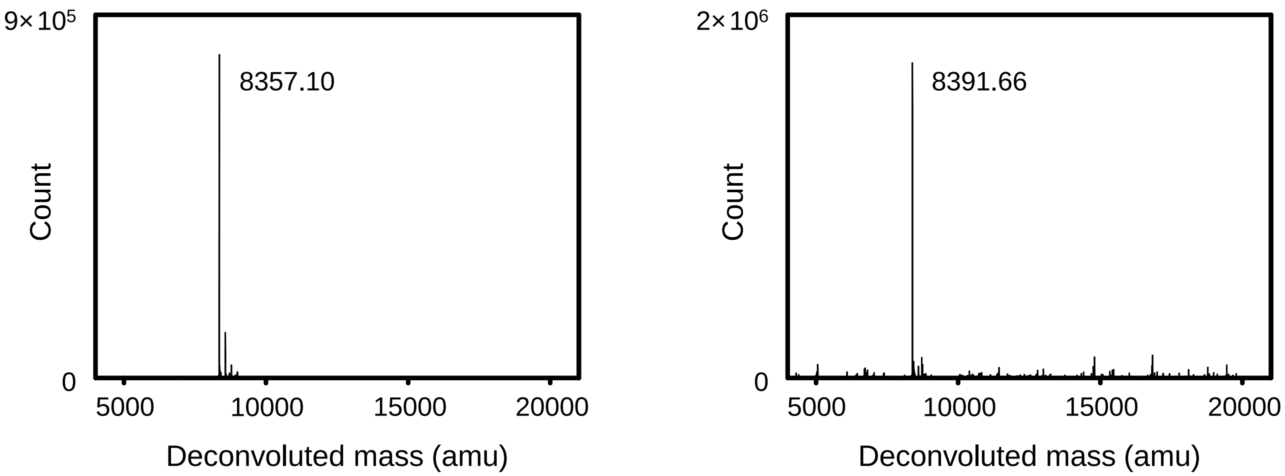

Supplementary Figure 8. Mass spectrometric analysis of recombinant peptides.
The maximum entropy deconvoluted spectra for rMaj-NPLP (left) and rMaj-pCHH-B (right) have been shown. Mass value has been shown in atomic mass units (amu). The N-terminal Ala residue of native Maj-NPLP has been substituted with a Gly residue in rMaj-NPLP because of the insertion of HRV 3C protease recognition site (see **Supplementary Figures 1 and 2**), and therefore, its calculated mass value is 8356.7 (8368.7 minus 12 for 6 disulfide bonds). Similarly, the N-terminal Gln residue has been substituted with a Gly residue in rMaj-pCHH-B which has a calculated mass value of 8391.6 (8397.6 minus 6 for 3 disulfide bonds).

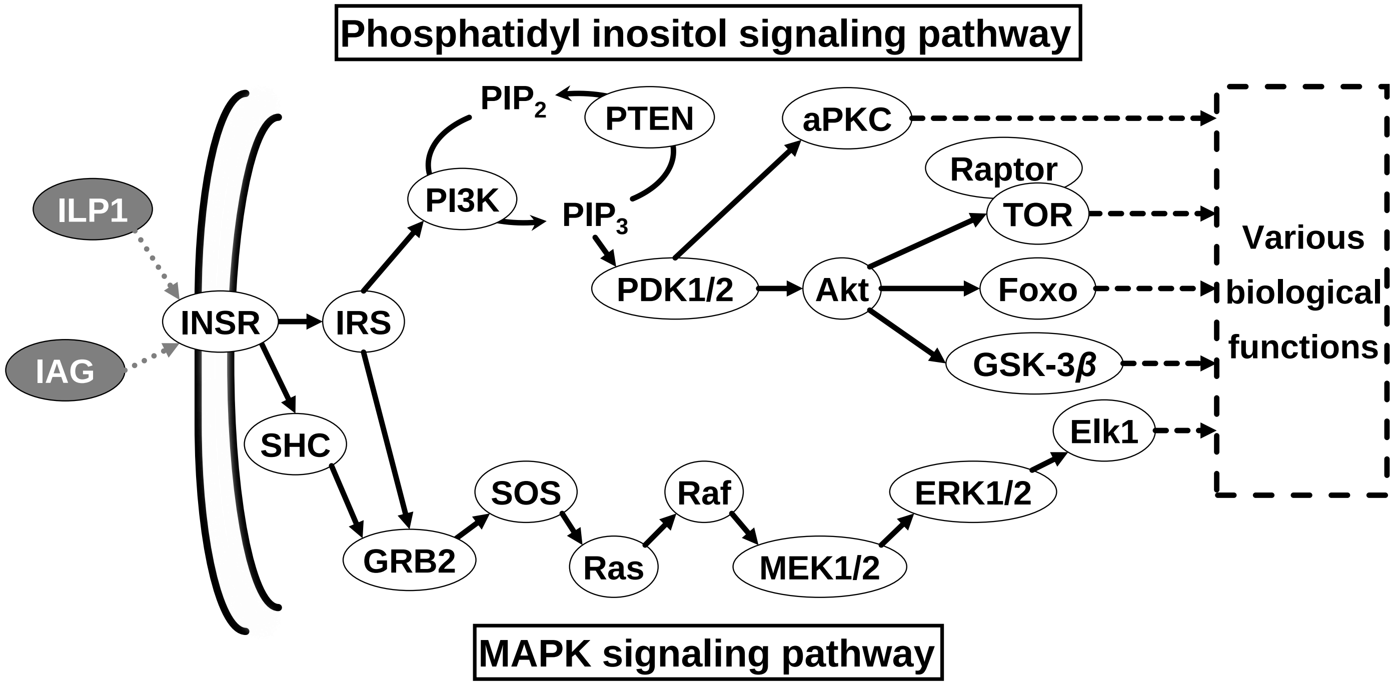

Supplementary Figure 9. Major components of the insulin signaling pathway found in the ovarian transcriptome.

Major components constituting insulin signaling pathway have been shown. Corresponding contig numbers are as follows; INSR: shown in Table 1, IRS: N17523, PI3K: N19194, PTEN: N15847, PDK1/2: N11361, aPKC: N13130, Akt: N11794, Raptor: N18832, TOR: N20179, Foxo: N09528, GSK-3β: N11687, SHC: N16759, GRB2: N11939, SOS: N14185, Ras: N30806, Raf: N14385, MEK1/2: N14775, ERK1/2: N09451, Elk1: N11680.
